# Recent Advances in Three-Dimensional Stem Cell Culture Systems and Applications

**DOI:** 10.1155/2021/9477332

**Published:** 2021-10-11

**Authors:** Xiaowen Wu, Junxiang Su, Jizhen Wei, Nan Jiang, Xuejun Ge

**Affiliations:** ^1^Shanxi Province Key Laboratory of Oral Diseases Prevention and New Materials, Shanxi Medical University School and Hospital of Stomatology, Taiyuan 030001, China; ^2^Central Laboratory, Peking University School and Hospital of Stomatology & National Engineering Laboratory for Digital and Material Technology of Stomatology & Beijing Key Laboratory of Digital Stomatology, Beijing 100081, China

## Abstract

Cell culture is one of the most core and fundamental techniques employed in the fields of biology and medicine. At present, although the two-dimensional cell culture method is commonly used *in vitro*, it is quite different from the cell growth microenvironment in vivo. In recent years, the limitations of two-dimensional culture and the advantages of three-dimensional culture have increasingly attracted more and more attentions. Compared to two-dimensional culture, three-dimensional culture system is better to realistically simulate the local microenvironment of cells, promote the exchange of information among cells and the extracellular matrix (ECM), and retain the original biological characteristics of stem cells. In this review, we first present three-dimensional cell culture methods from two aspects: a scaffold-free culture system and a scaffold-based culture system. The culture method and cell characterizations will be summarized. Then the application of three-dimensional cell culture system is further explored, such as in the fields of drug screening, organoids and assembloids. Finally, the directions for future research of three-dimensional cell culture are stated briefly.

## 1. Introduction

Cell culture is one of the most important and fundamental techniques in the fields of biology and medicine. It involves extracting cells from biological tissues, simulating the survival environment *in vivo* to ensure their growth and reproduction, and maintaining their main structures and functions under sterile conditions with suitable temperature, pH, and adequate nutrient conditions.

At present, cell culture methods *in vitro* include two-dimensional (2D) adherent culture and three-dimensional (3D) spherical culture [1], with the former being most typically used. In this method, a glass or polystyrene dish provides mechanical support for the cells, and the supplies of exogenous nutrients and the removal of metabolites are kept under the same conditions. The conditions are well controlled, and the cells are easy to be observed and collected. However, the 2D method has its drawbacks, as it fails to mimic complex cell microenvironment.

*In vivo*, most cells interact with neighbouring cells and the extracellular matrix (ECM) to form a complex communication network of biochemical and mechanical signals, which is the basis for maintaining cell normal functions [2]. Oxygen, hormones, and nutrients can be transported between cells, metabolic waste can be removed from cells, and cells can move in response to mechanical or chemical stimuli [3]. With 2D culture, cells grow in a limited space and thus being subject to contact inhibition. Thus, cell proliferation is slower, and cell morphology and cell function are also changed [4]. For example, stem cells are prone to lose self-renewal ability, become senescence, or differentiate into bone cells or adipocytes spontaneously [5]. Long-term cultures will gradually lead to tissue specificity lost [6], observed differences between the results of *in vitro* cell culture and *in vivo* animal experiments. Therefore, understanding how to better simulate the physiological environment under *in vitro* conditions is very important for medical research [7].

Continuous advancements of technology have yielded higher requirements for cell culture models, leading to the development of 3D cell culture. Compared to 2D culture, 3D culture is closer to the organism in structure and function and more accurately simulates the microenvironment of cells *in vivo* [1, 3, 8]. This 3D cell culture can affect cell growth and proliferation, promote the self-renewal of stem cells, and inhibit their differentiation. In addition, like cells *in vivo*, 3D culture is better for the transfer of molecules between cells and between cells and the ECM, nutrients uptake, gas exchange, and metabolic waste discharge in a balanced way [9, 10]. Therefore, to maintain the original characteristics of cells and better realistically simulate the state of the cells *in vivo* [11], researchers have developed a variety of 3D culture systems. For drug development, 3D culture has become a bridge between 2D culture and animal experiments [12]. In this review, we will first summarize the research states of 3D culture methods, with or without scaffold support (Table 1). Then, the applications of 3D culture will be assessed. In particular, we lay emphasis on the cutting-edge applications, for example, organoids and assembloids.

## 2. Three-Dimensional Stem Cell Culture Systems

**Figure 1:**
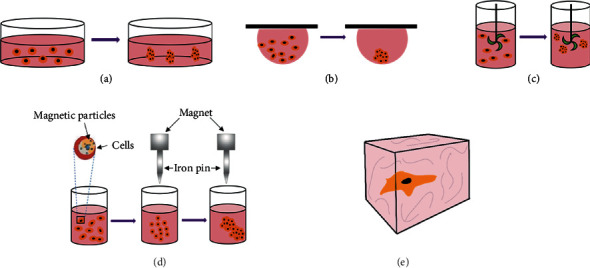
Scheme of 3D cell culture systems: (a) liquid overlay culture; (b) hanging drop culture; (c) rotating bioreactor culture; (d) magnetic suspension culture; (e) scaffold-based culture (hydrogel).

3D cell culture technology, which refers to the cocultivation of carriers with 3D structures of different materials and various types of cells in vitro, so that the cells can migrate and grow in the 3D structure of the carrier to form a 3D cell-carrier complex, can be divided into scaffold-free and scaffold-based culture systems [13], with both types having their own applications in different studies (Figure 1).

### 2.1. Scaffold-Free Culture System

This culture system does not have a supporting structure for cell adhesion, growth, and diffusion, leading to cells in the culture media to aggregate into tissue-like spheres called spheroids. These spheroids form their own ECM, without the need for exogenous scaffold or matrix. The ECM includes glycosaminoglycans, proteoglycans, structural proteins, adhesion proteins, and other macromolecular substances, which affect a series of activities such as cell shape, metabolism, function, migration, proliferation, and differentiation [14].

#### 2.1.1. Liquid Overlay Culture

The liquid overlay technique is one of the simplest and most economical 3D cell culture methods [15]. This method relies on materials that inhibit cell adhesion to the bottom of the cell culture vessel, such as agar and agarose, HEMA, or ultralow attachment plates [16]. The intercellular force is greater than between cells and the material surface [17], and this force between cells can spontaneously aggregate to form cell spheres within 24–72 hours [18–20]. Corning ultralow attachment microplates are commonly used, which are characterized by a covalently bonded hydrogel layer that minimizes cell adhesion, protein adhesion, and cell activation, with spontaneous cell assembly relying on self-secreted ECM [21].

#### 2.1.2. Hanging Drop Culture

The hanging drop culture is one of the most widely used methods of 3D cell culture and relies on the cell's own gravity to make individual cells aggregate to form 3D spheres [22, 23]. To perform this method, one drops the cell suspension droplets on the inner lid of the tissue culture dish, with the droplet volume being 10–20 *μ*L [24] and containing approximately 50–500 cells. Following this, the lid is turned upside down, and the droplets are fixed in place by surface tension. The microgravity environment of each droplet condenses the cells, forming a single sphere at the tip of the droplet and proliferating [25]. The size of the cell sphere can be controlled by adjusting the cell density of the cell suspension [26]. The obtained cell spheres are densely packed and uniform in morphology, forming spherical cells of the same size [27]. Additional advantages of this method include its low cost, convenient operation, high production efficiency, and coculturing of different types of cells. However, the disadvantages are that the volume of the cell suspension cannot be more than 30 *μ*L; otherwise, the droplets may fall. Additionally, workload is intensive and mass production is therefore difficult [2]. Since the cell suspension is small and can evaporate easily, changing cell culture medium is too difficult to maintain long-term cell culture. The 384-hanging drop array provides improvements to the hanging drop culture method. The design of the reservoir structure effectively reduces the evaporation of small-volume hanging drops. Part of the culture medium can be changed, so that the cell spheroids can be cultured for a longer time. Mass production of 3D spheres can be used for basic biological research [26].

#### 2.1.3. Rotating Bioreactor Culture

In this method, the high-density cell suspension is placed in the bioreactor, and the cell suspension is kept in motion by rotating and agitate so that the cells cannot settle and adhere to the substrate, therefore maximizing the contact between cells to form 3D spheres [18]. The system includes a container for cell culture and a continuously stirred impeller to ensure cell suspension and medium mixing. The flow of liquid not only prevents the adhesion of cells but also ensures the uniform distribution of various nutrients and oxygen, which is conducive to the formation and metabolism of 3D cell spheres. This method is relatively simple and can produce a large number of spheres in a short time. Cell culture using this method is simple and easy to mass production, the dynamic culture aids in nutrient transportation, and the spheroids are easy to obtain. However, the disadvantages are obvious in this system. The foam and shear stress of the fluid generated during the stirring process may cause damage to the cells, 3D cell spheres vary in size, and the special equipment is indispensable. Studies have shown that rotating cell culture can induce osteogenic differentiation of human bone marrow mesenchymal stem cells [28]. The possible reason is rotating culture which is more similar to the in vivo cell environment, is more conducive to bone formation, and promotes earlier osteocalcin synthesis and calcium deposition.

#### 2.1.4. Magnetic Suspension Culture

Magnetic suspension culture is a method that uses magnetic nanoparticles (such as iron oxide or gold nanoparticles) and magnetism to gather single cells into 3D spheres [29]. Cells are incubated with magnetic nanoparticles overnight to make them magnetic; then, the magnetic cells are extracted and recultured [30]. The magnetic field is applied during the cell culture process, and the cells gather into 3D spheres at a height whereby the magnetic force and gravity balance [31]. Spheroids can be formed rapidly within five minutes, have repeatability and size stability, and can be extracted and transferred by magnetic tools [24]. This method allows for coculturing of a variety of cells [32, 33]; however, the potential impact of nanoparticles on cell signaling and function is a limitation of this approach [34].

#### 2.1.5. Chemical Reagent Culture

Chemical reagent culture is a culture method that uses special chemical reagents to make cells self-assemble to form 3D spheres. Chen et al. found that due to difference in human or bovine serum albumin batches, the experimental results were inconsistent before and after culturing stem cells with TeSR medium. Subsequently, the components in TeSR media were studied. After sequentially screening, they finally developed a practical, determined, and albumin-free TeSR-E8 media, containing eight ingredients, namely, DMEM/F12, insulin, selenium, transferrin, L-ascorbic acid, FGF2, TGF*β*, and NaHCO_3_ [35], which is suitable for stem cell culture. Zhao et al. have verified that human mesenchymal stem cells with a chemically defined serum-free TeSR-E8 medium can spontaneously assemble into 3D spheres. And the research found compared to 2D, the stemness of 3D cells is enhanced, which increases the treatment efficiency of endotoxemia mice and reduces mortality [6].

### 2.2. Scaffold-Based Culture System

Natural ECM has poor mechanical properties and high sensitivity to enzymes, which limits its application potential [36]. In recent years, with the advancement of biomaterial technology, scaffolds composed of artificial ECM are the most commonly used material and can simulate the complex 3D structure and main characteristics of living tissues. The function of the scaffold is to provide a spatial living environment for cells and enhance their adhesion, proliferation, and secretion of cytokines. Furthermore, this scaffold can promote interactions between cells and between cell and the ECM and further affect the shape of cells, metabolism, function, migration, proliferation, and differentiation [37]. Moreover, it also serves as a medium for the diffusion of soluble factors.

3D cell culture scaffolds can be divided into two types according to the source of materials: natural material scaffolds and synthetic material scaffolds [38], including hyaluronic acid, collagen, polylactic acid, and polyethylene glycol. Among them, hydrogel is one of the most widely used materials for 3D culture [39]. There are natural polymer hydrogels and synthetic polymer hydrogels [40]. Hydrogel has a network structure with a large number of hydrophilic groups, which can hold a large amount of water. The network structure of the hydrogel allows nutrients and oxygen to flow in and out freely, and the cells in it can be adequately nourished [41]. At the same time, it can also cross-link bioactive factors to regulate cell growth and differentiation, making it an excellent substitute for ECM.

#### 2.2.1. Natural Polymer Hydrogel

Natural polymer hydrogels are mainly natural materials supplemented by other biological materials or molecules [42]. Natural materials are obtained from animal, plant, or human tissues or cells, including hyaluronic acid [43], collagen [44], fibrin [45], silk fibroin [46], alginate [47], chondroitin sulfate [48], gelatin [49], and agarose and chitosan sugar [50]. They individually or mutually aggregate to form a 3D network structure similarly to the organism under certain conditions.

Natural polymer materials have limited mechanical properties; the composition of ECM of human or animal is uncertain. Therefore, there may be pathogen risks and inconsistencies between batches [13, 51]. Natural polymers usually show good biocompatibility, sensitivity to the environment, low toxicity, and cell adhesion sites. Besides, wide source and low price are also its outstanding advantages. Furthermore, these natural materials have their own advantages, and their combination shows excellent performance. For example, some researchers have combined gelatin and polysaccharides to form gel scaffold, which takes advantage of the therapeutic and regenerative properties of gelatin and the mechanical properties of polysaccharides. Composite applications provide a promising method for the development of superior biomaterials [42]. The chitosan-alginate-gelatin composite hydrogel can promote the chondrogenic differentiation of hMSCs and contribute to cartilage regeneration in patients with related cartilage diseases [52]. Some researchers put hepatocyte-like cells derived from human pluripotent stem cells into the widely used animal-derived hydrogel Matrigel, which is a plant-derived nanocellulose hydrogel in agarose microporous 3D culture plates. These cells can all form 3D spheres and accelerate the liver maturation of hepatocyte-like cells, which further shows that the hydrogels of plant origin and animal origin have the same functions, but the former can avoid disadvantages such as endotoxin and batch-to-batch differences [53].

#### 2.2.2. Synthetic Polymer Hydrogel

Synthetic polymer scaffold materials include polylactic acid (PLA) [54], polyethylene glycol (PEG) [55–57], polycaprolactone (PCL) [58], polylactic acid glycolic acid (PLGA) [59], poly L-lactic acid (PLLA) [60], and polyglycolic acid (PGA) [61]. These polymers are cross-linked to form a hydrogel, which can be used as a 3D cell culture platform. This inert gel has a clear chemical composition, high reproducibility, high mechanical strength [62], simple processing and manufacturing, and greater predictability of results and higher versatility. Thus, the hydrogel possesses broad application prospects in tissue engineering scaffold materials [51]. Unfortunately, synthetic hydrogels usually do not have cell adhesion sites [13], integrin-binding peptides, or growth factor binding sites. And ECM degradation protease domains that promote cell-ECM cross-linking are required [7], making the construction process relatively complicated. Additionally, the shortcomings of poor biocompatibility, poor toughness, and slow water absorption limit their direct application in the field of cell culture scaffolds and therefore require continuous research and improvement.

Generally speaking, it is difficult for a single type of material to meet the requirements of cell culture scaffold materials. As such, combining several single materials through a suitable method and comprehensively considering the advantages and disadvantages of each of the materials to form a composite material can achieve good effects [63]. Due to natural material excellent water absorption performance, strong biocompatibility, low cost, and abundance, the inclusion of cell adhesion sites comprising natural materials and the adjustable mechanical strength of synthetic materials makes for an ideal combination for preparing 3D cultured hydrogel. Researchers have developed a collagen-bioceramic composite hydrogel that can promote the osteogenic differentiation of hADSCs, offering a new approach for the treatment of bone defects [64]. Some researchers have also used gelatin-methacryloyl hydrogel, as the fusion of biological, and biological manufacturing methods can accelerate the clinical transformation of tissue repair [65]. However, the mechanism of materials in regulating 3D culture cell functions and behaviours needs further investigation.

## 3. Applications

### 3.1. Drug Screening

The preclinical screening process of therapeutic drugs usually includes the process from 2D cells, evolving from animal models to clinical trials [66], with only a few drugs ultimately passing clinical trials for approval by regulatory agencies to enter the market [67]. One possible reason is that there are differences in tissue structure between cells and organs, as well as differences in cell growth patterns [68], with inherent differences between humans and animals. Additionally, animal models are expensive, time-consuming, and raise ethical issues [24]. At the same time, the increase in drug compounds and the requirements for high-throughput screening have slowed the progress of drug research.

In view of the above, it is necessary to develop more effective preclinical screening methods to accelerate the process of determining the failure of new drug research and development, as timely termination will cause a reduction in waste. 3D cell culture may be the best candidate, as the emergence of 3D culture models has greatly improved cell-based drug screening through the identification of toxic and ineffective substances in the early stages of drug discovery [69], bridging the gap between 2D cell analysis and animal experiment results and reducing the drawbacks caused by 2D culture [68]. The experimental uncertainty reduces the cost of drug development and achieves more effective drug screening [70]. Although the accepted standard for *in vitro* drug screening and *in vivo* toxicity studies is still the 2D cell culture model [70], the 3D culture is expected to become an effective tool in the drug development process.

In the screening of cancer drugs, the 3D culture method possesses special significance. On the one hand, it provides an *in vitro* model similar to *in vivo* tumors [71]. On the other hand, normal cells around the tumor can affect the sensitivity of tumor cells to drugs. Researchers have confirmed that using a 3D coculture model can accelerate the screening of insulin-resistant diabetes drugs, and spherical coculture can be used for tumor drug screening [72].

### 3.2. Organoids

Organoids are the cell-derived *in vitro* 3D-culture organ models, with pluripotent stem cells or progenitor cells of specific tissues forming similar tissues of corresponding organs [73]. They have the ability of self-renew and self-organization and maintain the characteristics of physiological structure and function of the tissues from which they originate [74]. The development of the 3D organoid culture system has presently been acknowledged as a major technological advancement in the field of stem cells [75].

Although 2D cells are widely used in biomedical researches for a long time, they are generally regarded as pure physical contact between cells, lacking in tissue structure and complexity [76]. Compared with 2D cell culture, organoids have more abundant cell types [77], closer behaviours to physiological cells [78], more stable genomes [73], and more suitable for biological transfection [79]. At present, a variety of 3D organoid models have been successfully constructed, such as optic cup/retinal organoids [80], brain organoids [81], intestinal organoids [82], kidney organoids [83], gastric organoids [84], liver organoids [85], pancreatic organoids [86], lung organs [87], vascular organoids [88], heart organoids [89], and bladder organoids [90].

**Figure 2:**
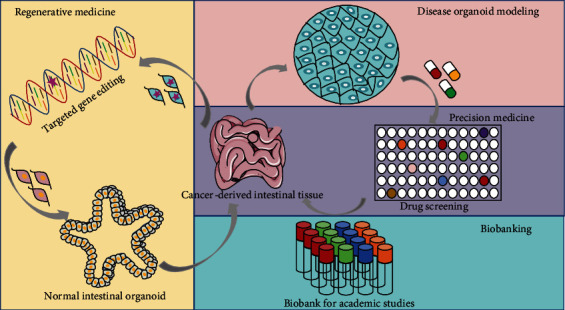
Multiple applications of organoid technology: (1) regenerative medicine; (2) disease modeling; (3) precision medicine; (4) biobanking.

As a 3D culture system that simulates the structure and function of organs *in vitro*, with the recognition, organoids are widely used in disease modeling, biobanking, precision medicine, and regenerative medicine [91] (Figure 2).

#### 3.2.1. Disease Modeling

Mounting achievements have provided convincing evidence for organoid application on disease models, such as tumors, developmental diseases, and infectious diseases [77]. Compared with the 2D culture system, 3D organoids help to clarify the development, homeostasis, and pathogenesis of diseases and provide possible new methods for the diagnosis and treatment [92].

Tumors are recognized as the primary cause of human death globally [93]. Composed of a variety of different cells, tumors develop as a result of complex intercellular interactions between cells and between cells and the ECM in a 3D environment [94]. Accordingly, 3D spheroids simulate tumor behaviour more effectively than conventional 2D cell cultures because spheroids are very similar to tumors [95]. These spheroids contain surface-exposed and deeply buried cells, proliferating and nonproliferating cells, and well-oxygenated and hypoxic cells [96], rendering them superior to 2D cells in terms of hypoxia [13], dormancy, antiapoptotic characteristics, and drug resistance [97]. Due to the complexity of tumor development, traditional 2D cultures cannot simulate the 3D microenvironment in which tumor cells reside and [98], as such, may provide misleading results on the predicted response of tumor cells to antitumor drugs [67]. Therefore, the tumor organoid model is acknowledged in cancer research.

Some characteristics of tumor organoids prove that it is suitable as a model for tumor research. Tumor organoids, which can be formed by using 3D culture technology, are the cultivation of tumor stem cells *in vitro*, maintaining the functions of the original stem cells and continuously dividing and differentiating to form microtumor tissues that are similar in space and structure to the source organ tissues, genes, structures, and functions [77, 99]. These organoids reproduce the *in vivo* characteristics and heterogeneity of the primary tumor and require a short time for formation and stable passage. They can be used for the study of tumorigenesis and development matrix, drug screening, individualized treatment, etc. [74]. 3D culture allows coculturing of multiple types of cells [69]. Some researchers have proposed that the existing cell culture methods are not enough to study fibroblasts and their interaction with cancer stem cells. They have found that fibroblasts promote the stemness of cancer stem cells under 3D environment [100] and their interaction affected cell invasion and metastasis [101]. The impact of tumor ECM on tumor progression has always been a hot issue for researchers [102]. 3D organoids provide tumor cells with a microenvironment consistent with *in vivo*, which is expected to find new tumor treatments.

Organoid models are used to study developmental diseases, especially brain organoids. Some researchers have developed a brain organoid model derived from human pluripotent stem cells, which can summarize the characteristics of human cerebral cortex development and can even be used to simulate microcephaly that is difficult to reproduce in mice. It also proved that the premature differentiation of neurons in the patient's organoids can be the cause of microcephaly [103]. Brain organoids have also been used to study Zika virus, which can preferentially infect neural progenitor cells and reduce their proliferation and viability. This may be an important cause of head deformities caused by Zika virus [104].

Organoids can also be used to simulate host-microbe interactions. With the emergence of various types of organoid models, the study of microbial infections will help to better understand the pathogenic mechanism and then find the best treatment strategy. Schlaermann et al. have established a powerful and quasi-immortal 3D organoid model, which is believed to be useful for future research aimed at understanding the underlying mechanisms of human gastric infections, mucosal immunity, and cancer [105]. Some organoid models have been applied to the study of microbial pathogenesis, for example, researchers used a kidney organoid model to study the mechanism of Shiga Toxin Type 2's renal cell toxicity [106]. Following the COVID-19 outbreak, Bing Zhao and Xinhua Lin's team used human organoids to study the molecular mechanisms of SARS-CoV-2 infection and liver damage, providing important tools for the study of new coronavirus cell tropism, pathogenic mechanisms, and subsequent drug development [107].

#### 3.2.2. Biobanking and Precision Medicine

For cancer research in the past, 2D culture is the most commonly used model in vitro for high-throughput drug sensitivity tests and correlating them with changes in the genome [108]. However, 2D culture often causes lost genomic characteristics of the original donor and does not maintain individual heterogeneity, which makes it difficult to accurately predict the sensitivity of specific patients to specific drugs. The human-derived tumor xenograft models can solve this problem well. The orthotopic tumor from patients can be transplanted into an immunodeficient animal, which can maximize the preservation of the heterogeneity of the donor [109]. However, the establishment of the 3D model is time-consuming and requires high costs, which is not conducive to high-throughput drug screening. Besides, organoids can be expanded indefinitely and cryopreserved [110]. Due to the above advantages, the creation of organoid biobanks becomes possible.

In 2015, Van De Wetering's team established a colorectal cancer organoid biobank for the first time [111]. It can be used for the study of the genome and its functions at the individual level of colorectal cancer patients and has the characteristics of short time-consuming and high-throughput, which cannot be achieved by traditional cell line models and human-derived tumor xenograft models. And because organoids can well retain the heterogeneity of donor tissues, they can be used for high-throughput and high-sensitivity drug sensitivity testing and make personalized treatment plans for colorectal cancer patients, which have high application value.

Subsequently, many different tumor organoid biobanks were established, including stomach, liver, pancreas, breast, prostate cancer, lung cancer, glioblastoma, and bladder cancer. As researcher's interest in the use of organoids for disease modeling grows, biobanks will soon expand beyond cancer, such as intestinal and lung organoids for cystic fibrosis patients [112] and for liver organoids of patients suffering from various metabolic diseases [113]. Scientists strive to create a biobank of organs of healthy and diseased patients as a renewable resource that can be used by researchers around the world.

Organoids through drug screening and drug safety testing provide a unique opportunity for precision medicine. A number of drug development failures in clinical trials are partly due to insufficient evaluation of drug toxicity in the preclinical testing phase. The emerging 3D organoid technology can correctly assess the toxicity of drugs, and it is possible to determine the best and most effective dose to kill tumor cells with minimal damage to normal tissues [114]. For example, liver and kidney organoids will be an excellent platform for evaluating potential drug-related liver and kidney toxicity [115, 116]. Before treatment, patient-derived organoids are used to screen drug responses *in vitro*, which have been proved to be valuable diagnostic tools [117].

#### 3.2.3. Regenerative Medicine

Organ transplantation is the most commonly used treatment for tissue and organ defects. However, this method has several shortcomings, such as insufficient donor tissue and a cumbersome process of donor selection, a risk of infection, and immune rejection [10]. In recent years, due to the favorable biological characteristics of stem cells, such as high proliferative capacity, self-renewal ability, multidirectional differentiation potential, abundancy, easy selection of materials, and absence of ethical issues, stem cells have received increasing attention from researchers in the field of tissue engineering and regenerative medicine, which has great clinical application value [118]. The stem cell transplantation process includes stem cell isolation, culture, targeted induction, and gene modification, with a large number of stem cells expanded *in vitro* or constructed into tissues and organs before being implanted in the body for treatment of clinical diseases [119].

As an *in vitro* model of tissue, organoids have attracted great attention in the field of regenerative medicine [120]. Providing appropriate 3D scaffolds and biochemical factors, cells derived from pluripotent stem cells can self-organize to form tissue-specific organoids, including optic cup [121], brain [103], intestine [122], liver [123], kidney [124], and pancreas [125].

After establishing the mouse intestinal organoids for the first time, Yui et al. injected the mouse colon organoids in the form of fragments into the mouse colitis model induced by sodium dextran sulfate. Later, the transplanted cells were observed to adhere to the injured intestinal area. Histological examination found that the graft formed a crypt-like structure in the colon of the recipient mouse. The functional test results show that the graft can maintain the intestinal epithelial barrier function [126]. Retinal tissue derived from mouse pluripotent stem cells (iPSC) has been transplanted by researchers into a mouse model of end-stage retinal degeneration, which showcased improvement of the vision of mice with end-stage retinal degeneration [127]. Additionally, researchers have used magnetic levitation to gather salivary secreting epithelial cells into 3D spheres to form salivary organoids, which can replace damaged salivary glands to secrete saliva under cholinergic stimulation [128]. Studies have also shown that, by rotating a bioreactor, bone marrow mesenchymal stem cells (BMSCs) form 3D spheres, which improves the osteogenic differentiation of (BMSCs) when implanted into a rat skull defect model to promote bone repair [28]. Moreover, researchers have made dental pulp stem cells (DPSCs) into 3D spheres without scaffolds through the action of heat-responsive hydrogel, and these spheres were introduced into human root canals as well as implanted under the skin of immune-deficient mice. After 6 weeks, a vascularized pulp-like tissue formed in the root canal [129].

These studies demonstrate that stem cells are induced to differentiate into specific tissues and organs, which can replace or repair damaged organs, and thus have broad prospects in the field of regenerative medicine.

The widespread application of organoid technology in the research community is still in its infancy, but as a tool, organoid technology has great potential, including developmental biology, disease pathology, cell biology, regeneration mechanisms, precision medicine, drug toxicity, and drug efficacy test. Organoid technology has unique and powerful characteristics that can completely change the traditional in vitro research tools used to simulate human development and diseases. However, the current organoid technology has a fundamental limitation, that is, it cannot simulate the mature structure of the organ and lacks the microenvironment within the tissue. In addition, there is a shortage of critical interactions between various cells in human tissues. These limitations have been regarded as the main problem for accurately simulating various refractory diseases including cancer.

### 3.3. Assembloids

The complexity of human organs has been difficult to study due to the dynamic interaction between numerous cell types and specific spaces. Organoids can be manipulated as a tool for studying development or disease; however, they reflect particular cell characters of the whole organ with inhomogeneity. In order to reflect the complicacy human tissues, the assembling of organoids is constantly increasing. Assembloids are organoids with spatial tissue structure produced by a variety of cell types [130]. This new type of microorgans can surpass organoids and is closer to real human tissue in structure and function [131].

Kim et al. constructed assembloids that simulate tissue regeneration and cancer for the first time [90]. They created multilayer bladder assembloids combining bladder stem cells with stromal, these assembloids were comprised of three compartments well organized to form bladder-like architectures. It contained multilayered urothelium and thick connective stroma, surrounded by a muscle layer. These researchers found that the cell composition and gene expression of these assembloids at the single-cell level are exactly the same as those of mature adult organs, and they mimic the in vivo regeneration response of normal tissues to injury. They also developed patient-specific tumor assembloids, which perfectly mimic the pathological characteristics of tumors in the body.

**Figure 3:**
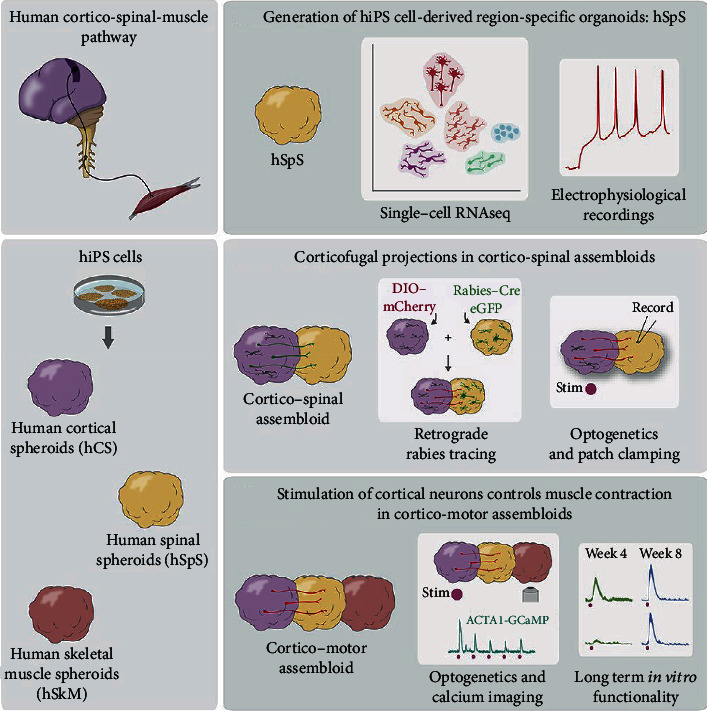
The assembly of 3D cultures derived from hiPS cells resembling the cerebral cortex, hindbrain/spinal cord, and skeletal muscle forms neural circuits that can be readily manipulated to model cortical control of muscle contraction in vitro. Reprinted with permission from [132], Copyright 2020 Elsevier.

In a new study, researchers from the Stanford University School of Medicine have assembled a working model of the human neural circuit responsible for autonomous movement for the first time in the scientific community [132]. They used human pluripotent stem cells to generate the three components of the neural circuit, including human cortical spheroids, human spinal spheroid, and human skeletal muscle spheroids, and let them assemble together in a dish. Glutamate uncaging or optogenetic stimulation of cortical spheroids triggers robust contraction of skeletal muscle spheroids, and assembloids are morphologically and functionally intact for up to ten weeks postfusion (Figure 3). This progress is expected to accelerate research on various neurological diseases.

Assembloids are powerful tools to unveil inaccessible aspects of neurobiology. To study how the human cortex-striatum pathway and its dysfunction can lead to neuropsychiatric diseases, Miura's team assembled the striatal organoids and cortical organoids to form cortical striatal assembloids [133]. The striatum is a brain structure that is the center of pleasant feelings and motivational behaviours. The assembloids may prove useful for studying the causes of schizophrenia, depression, and addiction. Martins et al. used spinal cord neurons and skeletal muscle cells derived from human pluripotent stem cells [134]. These cells self-organized to generate human neuromuscular assembloids. They successfully used neuromuscular assembloids to summarize the key aspects of the pathology of myasthenia gravis. This highlights the great potential of neuromuscular assembloids in simulating neuromuscular diseases in the future.

With the joint efforts of researchers around the world, it is believed that more and more assembloids will appear to provide more realistic models for the study of human tissues and organs, as well as pathological processes.

## 4. Conclusions

In recent years, 3D cell culture has emerged as a prominent culture technique. In comparison to 2D cell culture, the advantages are that it provides a 3D microenvironment in which cells complete proliferation, differentiation, movement, apoptosis, etc. 3D cell culture more accurately simulates the cell state in the human microenvironment to a large extent, so it has great developability. As such, it has potential applications in tissue engineering, regenerative medicine, drug development, toxicity testing, and organoid and assembloid formation. However, 3D culture technology is still in its infancy; its cost is still high, and because the culture conditions are not yet in the most optimal state, there is still a gap between the culture real situations in the body. Because the viability and differentiation of cells are limited, the present target of research is how to continue to improve the technology to make the 3D culture system closer to the actual environment of the human body, how to achieve an efficient and automated culture system while reducing the cost, how to better utilize the advantages of various materials in the design as well as the use of composite materials, etc. We believe that with the development and progress of tissue engineering technology, these problems, such as the interdisciplinary development of life sciences, engineering and materials science, and the unremitting efforts of scientific researchers, will be increasingly discussed in depth and, thus, will gradually be resolved.

## Figures and Tables

**Table 1:** A comprehensive summary of 3D cell culture methods.

Method	Culture cell types	Viability	Proliferation	Differentiation	Time length	Spheroid diameter (*μ*m)	Advantages	Disadvantages	Refs.
Liquid overlay	(1) MDPSCs; (2) ADSCs	Well-preserved		Osteogenic	-1 day	30-100	(1) Simple; (2) economical	Size of the spheroids cannot be controlled	[15–21]
Hanging drop culture	WJ-MSCs	Cell death rate was below 40%	Higher than 2D	Early osteogenic	Depend on cell types	Controllable	Uniformity	Volume cannot more than 30 *μ*L	[2, 22–27]
Rotating bioreactor	BMSCs	High level of viability		Osteoblastic	1 day	100–200	(1) Simple; (2) efficient	(1) Shear stress; (2) equipment	[18, 28]
Magnetic suspension	NIH3T3	99% cell viability	Exponential growth		5 minutes		(1) Repeatable ability; (2) size stability.	Potential impact of nanoparticles is uncertain	[24, 29–34]
Chemical reagents culture	ADSCs	High level of viability	Suppressed	Osteogenesis	3-4 days	50-200	(1) Simple; (2) practical; (3) special equipment unnecessary	Potential impacts of reagent are uncertain	[6, 35]
Alginate-PEG gels	mMSCs	Improves the viability of cell	Reducing cellular apoptosis	Osteogenic			Biocompatibility high mechanical strength	Early complicate	[36–57]

## Data Availability

No data were used, available upon request, or included within the article.
